# 

**Published:** 1902-06

**Authors:** 


*The following donations have been received since the publication of the List in March:*

*May 31st, 1902.*

| | |
|---|---|
| F. H. Edgeworth, M.B. (1) ... ... ... ... ... | 2 volumes. |
| The General Medical Council (2) ... ... ... ... | 1 volume. |
| L. M. Griffiths (3) ... ... ... ... ... ... ... | 7 volumes. |
| The Editor of *Illustrirte Rundschau der Medicinisch-Chirurgischen Technik* (4) ... ... ... ... ... | 1 volume. |
| The Medical Library, McGill University (5) ... | 2 volumes. |
| E. Merck (6) ... ... ... ... ... ... ... ... | 1 volume. |
| The Pathological Society of London (7) ... ... | 1 ,, |
| Arthur B. Prowse, M.D. (8) ... ... ... ... ... | 1 ,, |
| Sir James Sawyer (9) ... ... ... ... ... ... | 1 ,, |
| The Scholastic Trading Co., Bristol (10) ... ... | 2 volumes. |
| The Society for the Study of Disease in Children (11) ... ... ... ... ... ... ... ... ... | 1 volume. |

### FORTY-FOURTH LIST OF BOOKS.

The titles of books mentioned in previous lists are not repeated.

The figures in brackets refer to the figures after the names of the donors, and show by whom the volumes were presented. The books to which no such figures are attached have either been bought from the Library Fund or received through the *Journal.*

**Ballance and P. Stewart, C.A.** *The Healing of Nerves* ... ... ... ... ... 1901

**Ballet, A. Proust and G.** *The Treatment of Neurasthenia* (Tr. by P. C. Smith) ... ... ... ... ... ... ... ... ... ... ... 1902

**Beaunis, H.** ... ... *Le Somnambulisme provoqué* ... ... (1) 2e Éd. 1887

**Boas, I.** ... ... *Diseases of the Intestines* (Tr. by S. Basch) ... ... 1901

**Browne, G. B.** ... *The Harveian Lectures on Twenty-five Years' Experience of Urinary Surgery in England* ... ... ... (8) 1901

**Burghard, W. W. Cheyne & F. F.** *Manual of Surgical Treatment.* Part VI. 1902

*Catalogue of Lewis's Medical and Scientific Library.* Supplement, 1898—1901 1902

**Cheadle, W. B.** ... *Artificial Feeding and Food Disorders of Infants* 5th Ed. (Ed. by F. J. Poynton) ... ... ... ... 1902

**Cheyne and F. F. Burghard, W. W.** *Manual of Surgical Treatment.* Part VI. ... ... ... ... ... ... ... ... ... ... 1902

**Clark, J. W.** ... *The Care of Books* ... ... ... ... ... ... ... ... ... 1901

*Climates and Baths of Great Britain, The.* Vol. II. ... ... ... ... ... 1902

**Critchett, G.** ... *On Ulcers of the Lower Extremity* ... ... ... ... (3) 1849

**Edwardes, E. J.** ... *Small-Pox and Vaccination in Europe* ... ... ... ... 1902

*Encyclopædia Medica* (Ed. by C. Watson) ... ... ... Vols. IV.—X. 1900–02

**Finsen, N. R.** ... *Phototherapy* (Tr. by J. H. Sequeira) ... ... ... 1901

**Freyer, P. J.** ... *Stricture of the Urethra and Enlargement of the Prostate* 2nd Ed. 1902

**Gibson and [W.] Russell, [G. A.].** *[Physical Diagnosis* (Ed. by F. D. Boyd) 3rd Ed. 1902

**Hayward, J. W.**... *Protoplasm* ... ... ... ... ... ... ... .. ... 1902

**Henle, A.** ... ... *Mikulicz's Conservative Treatment of Tubercular Joint Disease* (Tr. by C. W. Cathcart) ... ... ... ... 1900

**Hood, W. P.** ... *The Treatment of Injuries* ... ... ... ... ... ... ... 1902

**Hughes, A. W.** ... *A Manual of Practical Anatomy.* Part III. (Ed. and completed by A. Keith). ... ... ... ... 1902

**Hutchison and H. Rainy, R.** *Clinical Methods* ... ... ... [2nd] Ed. 1902

**Jenner, E.** ... ... *On the Varieties and Modifications of the Vaccine Pustule* ... ... ... ... ... ... ... ... ... (3) 1806

**Knopf, S. A.** ... *Tuberculosis as a Disease of the Masses and How to Combat It* ... ... ... ... ... .. ... ... ... 1902

**Latimer, J.** ... ... *The Annals of Bristol in the Nineteenth Century* (concluded), 1887—1900 .. ... ... ... ... 1902

**Limbeck, R. R. v.** *The Clinical Pathology of the Blood* (Tr. from the 2nd German Edition by A. Latham and J. Nachbar) 1901

**Macnaughton-Jones, H. [et al].** *The Practitioners' Handbook of Diseases of the Ear and Naso-Pharynx* ... ... ... 6th Ed. 1902

**Mann, J. D.** ... *Forensic Medicine and Toxicology* ... ... 3rd Ed. 1902

**Moll, A.** ... ... *Christian Science, Medicine and Occultism* (Tr. by F. J. Rebman)... ... ... ... ... ... ... ... ... 1902

**Muirhead, C.** ... *The Causes of Death among the Assured in the Scottish Widows' Fund and Life Assurance Society from 1874 to 1894 inclusive* ... ... ... ... ... ... 1902

**Paget, Sir James.** *Selected Essays and Addresses* (Ed. by S. Paget) ... 1902

**Proust and G. Ballet, A.** *The Treatment of Neurasthenia* (Tr. by P. C. Smith) ... ... ... ... ... ... ... ... ... 1902

**Rainy, R. Hutchison and H.** *Clinical Methods* ... ... ... [2nd] Ed. 1902

**Ricord, [P.]**... ... *Lectures on Chancre* (Tr. by C. F. Maunder) ... (3) 1859

**Rieger, C.** ... ... *Der Hypnotismus* ... ... ... ... ... ... ... ... (1) 1884

**Russell, [G. A.] Gibson and [W.]** *Physical Diagnosis.* (Ed. by F. D. Boyd) ... ... .. ... ... ... ... 3rd Ed. 1902

**Sawyer, Sir J.** ... *Contributions to Practical Medicine* ... (9) 3rd Ed. 1902

**Stewart, C. A. Ballance and P.** *The Healing of Nerves* ... ... ... ... 1901

**Thomson, H. C.** ... *Acute Dilatation of the Stomach* ... ... ... ... ... 1902

**Treves, F.** ... ... *The Tale of a Field Hospital* ... ... ... ... (3) 1900

**Voselang, A.** ... *The Springs and Climate of Tarasp-Schuls-Vulpera, Engadine—Switzerland* ... ... ... ... ... ... 1902

**Walker, N.** ... ... *An Introduction to Dermatology* ... ... ... 2nd Ed. 1902

### TRANSACTIONS, REPORTS, JOURNALS, &c.

Alienist and Neurologist, The ... ... ... ... ... ... Vol. XXII. 1901

American Gynæcological and Obstetrical Journal, The ... Vol. XIX. 1901

American Journal of Obstetrics, The ... ... ... ... ... Vol. XLIV. 1901

American Journal of Ophthalmology, The ... ... ... ... Vol. XVIII. 1901

American Medical Quarterly, The ... ... ... ... ... ... Vol. I. 1900

American Practitioner and News, The ... Vols. XXXI., XXXII. 1901

Annales de la Société Belge de Chirurgie ... Tome IX. 1901

Annali di Ostetricia e Ginecologia ... Vol. XXIII. 1901

Archiv für Verdauungs-Krankheiten ... Bd. VI. 1900

Archives de Neurologie ... $2^{e}$ Sér., Tomes XI., XII. 1901

Archives of Pediatrics ... Vol. XVIII. 1901

Archives provinciales de Chirurgie ... Tome X. 1901

Association of American Physicians, Transactions of the ... Vol. XVI. 1901

Birmingham Medical Review, The ... Vols. XLIX., L. 1901

Bookseller, The ... 1901

Boston Medical and Surgical Journal, The ... Vol. CXLV. 1901

British Journal of Dermatology, The ... Vol. XIII. 1901

Brooklyn Medical Journal, The ... Vols. XIV., XV. 1900-01

Bulletin de l'Académie de Médecine ... $3^{e}$ Sér., Tomes XLIII., XLIV. 1900

Bulletin de l'Académie royale de Médecine de Belgique

$4^{e}$ Sér., Tome XV. 1901

Caledonian Medical Journal, The ... N.S., Vol. IV. 1901

Canadian Practitioner and Review, The ... Vol. XXVI. 1901

Centralblatt für Chirurgie ... 1901

Centralblatt für Gynäkologie ... 1901

Centralblatt für innere Medicin ... 1901

China Medical Missionary Journal, The ... Vol. XV. 1901

Cincinnati Lancet-Clinic, The ... Vol. LXXXVI. 1901

Clinical Journal, The ... Vol. XVIII. 1901

College of Physicians of Philadelphia, Transactions of the

3rd Ser., Vol. XXIII. 1901

Convocation of York—Report on Intemperance ... (3) 1874

Dermatological Society of Great Britain and Ireland, Transactions of the ... Vols. V.—VII. 1899—1901

Écho médical du Nord, Le ... 1901

Finska Läkaresällskapets adertonde allmänna Möte i Helsinfors, 19—21 September 1901, Förhandlingar vid ... 1902

Gazzetta Medica Lombarda ... 1901

Gazette de Gynécologie ... Tome XVI. 1901

Gazette des Hôpitaux ... 1901

Gazette des Hôpitaux de Toulouse ... 1901

Gazette hebdomadaire des Sciences médicales de Bordeaux Tome XXII. 1901

General Medical Council, Minutes of the ... (2) Vol. XXXVIII. 1902

Glasgow Medical Journal, The ... Vol. LVI. 1901

Grèce médicale, La ... 1901

HOSPITALS—

Royal Victoria Hospital, Montreal, Studies from the.

Vol. I., Nos. 1, 2 (5) 1901-02

Hospitals and Charities, Burdett's ... 1902

Illustrirte Rundschau der Medicinisch-Chirurgischen Technik ... (4) 1899

Indian Medical Gazette, The ... Vol. XXXVI. 1901

Intercolonial Medical Journal of Australasia, The ... Vol. VI. 1901

International Medical Magazine, The ... Vol. X. 1901

Johns Hopkins Hospital Bulletin ... Vol. XII. 1901

Journal de Neurologie ... Tome VI. 1901

Journal of Balneology and Climatology, The ... ... ... ... Vol. V. 1901
Journal of Cutaneous and Genito-Urinary Diseases ... ... Vol. XIX. 1901
Journal of Experimental Medicine, The ... ... ... ... Vol. V. 1900-01
Journal of Hygiene, The ... ... ... ... ... ... ... ... ... Vol. I. 1901
Journal of Laryngology, Rhinology, and Otology, The ... Vol. XVI. 1901
Journal of Mental Science, The ... ... ... ... ... ... Vol. XLVII. 1901
Journal of Nervous and Mental Disease, The ... ... Vol. XXVIII. 1901
Journal of the American Medical Association, The ... Vol. XXXVII. 1901
Journal of Tropical Medicine, The ... ... ... ... ... .. Vol. IV. 1901
Liverpool Medico-Chirurgical Journal, The ... ... ... Vol. XXI. 1901
Llangyfelach Rural District Council—Annual Report on the Llandilo-Talybont Division for 1901 ... ... ... ... ... ... ... ... 1902
Medical Age, The ... ... ... ... ... ... ... ... ... Vol. XIX. 1901
Medical Annual, The ... ... ... ... ... ... ... ... ... ... ... 1902
Medical News, The ... ... ... ... ... ... ... ... ...Vol. LXXIX. 1901
Medical Press and Circular, The ... ... ... ... ... [Vol. CXXIII.] 1901
Medical Record, The ... ... ... ... ... ... ... ... ...Vol. LX. 1901
Medical Review, The ... ... ... ... ... ... ... ... ... Vol. IV. 1901
Medico-Chirurgical Transactions ... ... ... ... ... Vol. LXXXIV. 1901
Merck's Annual Report [on the New Medicinal Preparations] for 1901 (6) 1902
Mercy and Truth ... .. ... ... ... ... ... ... ... ... ... Vol. V. 1901
Metropolitan Asylums Board—Report on the Bacteriological Diagnosis and the Antitoxic Serum Treatment of Cases admitted to the Hospitals of the Board, 1895-96 ... ... ... ... ... ... [N.D.]
Monthly Cyclopædia of Practical Medicine, The ... N.S., Vol. IV. 1901
Montreal Medical Journal, The ... ... ... ... ... ... Vol. XXX. [1901]
Münchener medicinische Wochenschrift ... ... ... ... ... .. ... 1901
New York Medical Journal, The ... ... ... ... ... ...Vol. LXXIV. 1901
Nord médical, Le ... ... ... ... ... ... ... ... ... Vol. VII. 1901
Occidental Medical Times, The ... ... ... ... ... ... Vol. XV. 1901
Odontological Society of London, Transactions of the N.S., Vol. XXXIII. 1901
Pathological Society of London, General Index to the Transactions of the, Vols. XXXVIII.—L., 1887—1899... ... ... ... (7) 1901
Philadelphia Medical Journal, The ... ... ... ... ... Vol. VIII. 1901
Polyclinic, The ... ... ... ... ... ... ... ... ... ... ... Vol. V. 1901
Post-Graduate, The ... ... ... ... ... ... ... ... ... Vol. XVI. [1901]
Practitioner, The ... ... ... ... ... ... ... ... ... Vol. LXVII. 1901
Progrés mêdical, Le ... ... ... ... ... 3e Sêr., Tomes XIII., XIV. 1901
Progressive Medicine ... ... ... ... ... ... ... ... ... Vol. I. 1902
Publishers' Circular, The ... ... ... (10) Vols. LXXIV., LXXV. 1901
Revue de Thêrapeutique mêdico-chirurgicale ... ... ... ... ... ... 1901
Revue gênêrale d'Ophtalmologie ... ... ... ... ... ... Tome XX. 1901
Revue hebdomadaire de Laryngologie, d'Otologie et de Rhinologie Tome XXI. 1901
Royal Academy of Medicine in Ireland, Transactions of the Vol. XIX. 1901
St. Louis Medical and Surgical Journal, The ... Vols. LXXX., LXXXI. 1901
Sanitary Record, The ... ... ... ... ... Vols. XXVII., XXVIII. 1901
Scottish Medical and Surgical Journal, The ... ... ... ... Vol. IX. 1901
Sei-I-Kwai Medical Journal, The ... ... ... ... ... ... Vol. XX. 1901

Society for the Study of Disease in Children, Reports of the (11) Vol. I. 1901
Therapeutic Gazette, The ... ... ... ... ... ... ... Vol. XXV. 1901
Treatment ... ... ... ... ... ... ... ... ... ... ... ... Vol. V. 1901
Warmley Rural District Council—Annual Report of Medical Officer of Health ... ... ... ... ... ... ... ... ... ... ... ... 1901
West London Medical Journal, The ... ... ... ... ... ... Vol. VI. 1901
Wiener klinische Wochenschrift ... ... ... ... ... ... ... ... ... 1901

---

### MEETINGS OF SOCIETIES.

### Local Medical Notes.

**Bristol Royal Infirmary.**—Mr. W. Mair, M.B. (Edin.), has been appointed Casualty Officer.

**Bristol General Hospital.**—Mr. A. F. Martin, M.B. (Vict.), has been appointed House Surgeon, Mr. J. E. Sparks, M.R.C.S.,

Assistant House Physician, Mr. A. Coleridge, M.R.C.S., Casualty Officer, and Mr. C. Shaw, M.B. (Lond.), Casualty Officer.

**Bristol Public Health.**—The prevalence of some considerable amount of Smallpox in London during the winter 1901-2 might have led to the supposition that this would prove an especial source of danger to Bristol. But of the four introduced cases one only, in December, came from London; the other three, of which one was fatal, came from the United States, in parts of which smallpox has been seriously prevalent. The cases remained "samples," no extension from any of them taking place, possibly because the port and city are under the same control, so that no slip between conflicting authorities occurred. This is the crux of smallpox control in a vaccinated community, and the risk from occasional introductions of this disease is by no means great if early recognition and notification be secured. One case has also been nursed in the city hospital for the Long Ashton Guardians, a tramp who came from the Midlands.

Diphtheria.—Of far greater urgency, though favoured with less of public regard, has been the continued threatening aspect of diphtheria. For fifteen years previous to 1900 the mortality records of Bristol showed that she fortunately had much less than her due share of diphtheria mortality; and seven years' study of the bacteriological conditions accompanying diphtheria prevalence in the city has convinced me that this was due to the non-introduction into the city previous to that date of the virulent bacillus which has prevailed since 1900. The forms present before that date were morphologically different, and in practice were found to be subject to ready control. Since the introduction of the present prevailing bacillus into Bedminster early in 1900, however, the tendency to spread, and the high fatality of the disease, have been very marked. Bedminster and Knowle, which suffered severely for the first eighteen months, are now practically clear, but the disease has successively attacked one fresh district after another,—Redcliff, Hotwells, St. George,—showing in many cases well-marked school incidence. The method of control has been on the lines adopted since 1895 in this city, and similar to those used by Cobbett at Cambridge and Colchester, *i.e.*, dealing with the disease as a purely parasitic disease, personally spread, largely by the agency of mild and unrecognised cases affecting the throat or nose of contacts. Those cases especially which show the presence of the atypical forms of diphtheria, accompanied by little or no signs of clinical illness, have received particular attention, and the prohibition of school attendance, with the provision of out-patient local treatment for such cases, has been attended with the happiest results. Personal medical inspection of contacts in affected classes, or in invaded houses, while necessitating a very large amount of work, is essential, and would justify in all towns more complete medical organisation in the public health service, to replace some excess of non-medical sanitary inspecting power. The control of diphtheria is purely a question of skilled medical inspection, and this disease must of all others be dealt with according to Koch's axiom—by having regard to its special etiology. The special feature of the early part of 1902 was the implication of the Two Mile Hill Schools, containing 1,100 children; this outbreak seems, however, to be already under control.

